# Replacing nitrogen in mineral fertilizers with nitrogen in maize straw increases soil water-holding capacity

**DOI:** 10.1038/s41598-024-59974-9

**Published:** 2024-04-23

**Authors:** Xiaojuan Wang, Le Tian, Tianle Wang, Enhui Zhang

**Affiliations:** 1 Shanxi Institute of Organic Dryland Farming, Shanxi Agricultural University, Taiyuan, 030031 Shanxi People’s Republic of China; 2 College of Agriculture, Shanxi Agricultural University, Taigu, 030801 Shanxi People’s Republic of China; 3 State Key Laboratory of Integrative Sustainable Dryland Agriculture (in Preparation), Shanxi Agricultural University, Taiyuan, 030031 Shanxi People’s Republic of China; 4 Key Laboratory of Sustainable Dryland Agriculture (Co-Construction By Ministry of Agriculture and Rural Affairs and Shanxi Province), Shanxi Agricultural University, Taiyuan, 030031 Shanxi People’s Republic of China; 5 Shanxi Province Key Laboratory of Sustainable Dryland Agriculture, Shanxi Agricultural University, Taiyuan, 030031 Shanxi People’s Republic of China

**Keywords:** Maize straw, Mineral fertilizer, Soil equivalent pore, Soil water availability, Soil water constant, Soil water retention curve, Ecology, Plant sciences

## Abstract

Soil water-holding capacity decreases due to long-term mineral fertilizer application. The objective of this study was to determine how replacing mineral fertilizer with maize straw affected the soil water retention curve, soil water content, soil water availability, and soil equivalent pore size. Replacement treatments in which 25% (S_25_), 50% (S_50_), 75% (S_75_), and 100% (S_100_) of 225 kg ha^−1^ nitrogen from mineral fertilizer (CK) was replaced with equivalent nitrogen from maize straw were conducted for five years in the Loess Plateau of China. The Gardner model was used to fit the soil water retention curve and calculate the soil water constant and equivalent pore size distribution. The results indicated that the Gardner model fitted well. Replacing nitrogen from mineral fertilizer with nitrogen from straw increased soil specific water capacity, soil readily available water, soil delayed available water, soil available water, soil capillary porosity, and soil available water porosity over time. S_25_ increased field capacity and wilting point from the fourth fertilization year. S_50_ enhanced soil readily available water, soil delayed available water, soil available water, and soil available water porosity from the fifth fertilization year, whereas S_25_ and S_75_ increased these from the third fertilization year or earlier. Soil specific water capacity, soil readily available water, soil delayed available water, soil available water, soil capillary porosity, and soil available water porosity could better reflect soil water-holding capacity and soil water supply capacity compared with field capacity and wilting point.

## Introduction

Plants absorb nitrogen and water for growth and metabolism. Nitrogen in plants mainly comes from soil. The relationship between nitrogen fertilizer and water is interdependent, and they work together in all aspects of plant growth. Appropriate amount of soil moisture content can promote nitrogen migration and transformation, and improve nitrogen availability, which is beneficial. Applying an appropriate amount of nitrogen can improve soil fertility and provide sufficient nutrients for plants^1^. Water is the medium of nitrogen absorption and transport. Adequate water is beneficial to plant roots to absorb nitrogen and promote the effective use of nitrogen in plants. After nitrogen fertilization, soil microbial community structure changed and soil bacterial diversity decreased. Soil water status changed the composition and activity of the soil microbial community by affecting the transport of soil nutrients and soil properties^2,3^. However, a large amount of nitrogen that is not absorbed and utilized by crops is lost through volatilization, nitrification–denitrification, runoff and leaching^4,5^.

Straw is an important by-product of crop production and an important agricultural resource. How to understand the value of straw resources and the rational use of straw resources has become a focus of attention from all walks of life. As far as the current utilization of straw resources is concerned, returning to the field is the most important way of utilization. Straw incorporation increased soil organic matter^6^, and it significantly affected the physical, chemical and biological processes of soil, and had a significant effect on soil structure and function. Besides, straw incorporation improved soil structure^7^, enhanced soil nutrients^8^, and stimulated soil enzymatic activities^9^. The activities of soil hydrolases including β-1,4-xylosidase, β-d-cellulase and β-1,4-glucosidase were increased by straw returning in paddy fields^10^. Whereas long-term application of mineral fertilizer resulted in soil compaction^11^ and caused soil degradation and seriously affected soil health. Besides, due to the inefficient use of nitrogen in crop and animal production, a large amount of nitrogen is lost, resulting in various environmental problems, including soil acidification, eutrophication water pollution and climate change, which are threatening our ecological environment^12,13^. Therefore, replacing mineral fertilizers with maize straw is necessary.

Previous studies mainly focused on straw incorporation combined with mineral fertilizer^8,14^. Wang^15^ showed that long-term application of mineral fertilizer and wheat straw returning increased soil available porosity and soil water-holding capacity. Pang^16^ found that two-year rotary tillage plus one-year deep tillage with maize straw returning and nitrogen fertilizer application increased the total porosity in the 0–20-cm soil layer and soil capillary porosity in the 10–30-cm soil layer compared with three-year rotary tillage with maize straw returning and nitrogen fertilizer application. Li^17^ observed that reduction in 1/6 chemical nitrogen fertilizer plus 6000 kg ha^−1^ maize straw increased soil non-capillary porosity compared with 540 kg ha^−1^ chemical nitrogen fertilizer application in the second and third fertilization years.

However, little is known about the effect of replacing nitrogen provided by mineral fertilizer with equivalent nitrogen provided by maize straw on the soil water retention curve and soil water availability. Thus, a five-year field experiment of equivalent nitrogen provided by different rates of maize straw incorporation and mineral fertilizer was established. We hypothesized that replacing nitrogen provided by mineral fertilizer with equivalent nitrogen provided by maize straw would enhance soil porosity, thereby increasing field capacity and soil water availability, and decreasing wilting point. The objective of this study was to evaluate the effects of replacing nitrogen from mineral fertilizer with equivalent nitrogen from maize straw on the soil water retention curve, soil water content, soil water availability, and soil equivalent pore size.

## Materials and methods

### Site description and experimental design

A five-year field experiment was performed using loam (sand 39.8%, silt 31.1%, and clay 29.1%)^18^ under maize cultivation in 2016–2020 at the Dongyang Research Station of Shanxi Agricultural University, Jinzhong, Shanxi, China (37° 56′ N, 112° 69′ E; 800 m altitude). The mean annual air temperature was 9.8 °C. The mean minimum air temperature of the coldest month (January) was − 6.1 °C, and the mean maximum air temperature of the hottest month (July) was 28.1 °C. The experimental site was characterized by low and erratic rainfall with droughts occurring at different stages of maize growth. The long-term mean annual rainfall at the site was 430.2 mm and the mean annual evaporation was 1860.1 mm. The rainfall was 352.4, 308.0 and 572.1 mm during 2018, 2019 and 2020, respectively. Analysis of soil samples taken from the same experimental area in April 2016 showed that the top 20 cm of soil was characterized as follows: pH 8.4, soil organic matter 13.0 g kg^−1^, total nitrogen 1.3 g kg^−1^, total phosphorus 0.9 g kg^−1^, total potassium 27.1 g kg^−1^, available nitrogen 51.2 mg kg^−1^, available phosphorus 7.7 mg kg^−1^, and available potassium 176.4 mg kg^−1^.

The field experiment used a completely randomized block design with five treatments and three replicates in a 5 × 6 m plot. Nitrogen provided by maize straw instead of 0%, 25%, 50%, 75%, and 100% of 225 kg ha^−1^ nitrogen provided by mineral fertilizer were conducted in 2016–2020. The five treatments were as follows: (i) application of 100% of 225 kg ha^−1^ nitrogen provided by mineral fertilizer only (CK); (ii) application of 25% (56.25 kg ha^−1^) of 225 kg ha^−1^ nitrogen provided by maize straw in combination with 75% (168.75 kg ha^−1^) of 225 kg ha^−1^ nitrogen provided by mineral fertilizer (S_25_); (iii) application of 50% (112.50 kg ha^−1^) of 225 kg ha^−1^ nitrogen provided by maize straw in combination with 50% of 225 kg ha^−1^ nitrogen provided by mineral fertilizer (S_50_); (iv) application of 75% of 225 kg ha^−1^ nitrogen provided by maize straw in combination with 25% of 225 kg ha^−1^ nitrogen provided by mineral fertilizer (S_75_); and (v) application of 100% of 225 kg ha^−1^ nitrogen provided by maize straw only (S_100_). Maize straw was incorporated at a 0–15 cm soil depth in each experimental year in late October. The 105 kg ha^−1^ phosphorus provided by mineral fertilizer was applied to CK. Replacement treatments applied phosphorus provided by mineral fertilizer with 105 kg ha^−1^ minus the phosphorus content of maize straw incorporated into soil. The mineral nitrogen and phosphorus fertilizers were applied separately as basal fertilizers before sowing maize. Urea and monoammonium phosphate were also applied. In each experimental year, the Dafeng 30 maize variety was planted at a rate of 49,500 plants ha^–1^ in late April or early May and harvested in late September.

### Sampling and analysis methods

Soil samples used for measuring the soil water retention curve were collected with a cutting ring at the plow layer after maize harvest. The soil samples were saturated slowly (> 24 h), weighed, and finally put into the CR22N high-speed refrigerated centrifuge (Hitachi Co.) to perform the soil water retention curve measurements starting from full saturation at 20 °C. The soil sample weight was measured at 10, 30, 50, 80, 100, 300, 500, 800, 1000, and 1500 kPa. Subsequently, the soil samples were oven-dried at 105 °C for 24 h. The volumetric water content at different suction levels was calculated using the equation:

1 $$ \theta ~ = ~\frac{{V_{W} }}{V} = \frac{{W_{S} ~{-}~W_{o} }}{{\rho ~ \times ~V}} $$

where *θ* is the soil volumetric water content at a certain suction (cm^3^ cm^−3^), *V*_*W*_ is the volume of water of the soil sample at a certain suction (cm^3^), *V* is the volume of the soil sample with 100 cm^3^ (cm^3^), *W*_*S*_ is the soil sample weight under a certain suction (g), *W*_*o*_ is the soil sample weight after oven-drying (g), and *ρ* is the water density with 1 g cm^−3^ (g cm^−3^)^19^.

The Gardner model was used to fit the acquired data in Microsoft Excel 2016 (Microsoft Corp., Redmond, WA, USA) as follows^20^:

2 $$\theta = {A }\times {{S}}^{-B}$$

Soil specific water capacity was derived from formula (2), and it was defined as

3 $${C }= {{A}} \times{B }\times {{ S}}^{-({{B}}+1)}$$

where *θ* is the soil volumetric water content (cm^3^ cm^−3^), *S* is the soil water suction (kPa), *A* and *B* are dimensionless parameters related to the curve shape, and *C* is the soil specific water capacity (kPa^−1^)^21^.

The field capacity, soil volumetric water content at 600 kPa, and wilting point were calculated by the Gardner model at 33, 600, and 1,500 kPa, respectively^22^.

Soil readily available water, soil delayed available water, and soil available water content were defined as

4 $${\theta}_{{r}} \, ={ \, {\theta}}_{{f}} \, - \, {\theta}_{600}$$

5 $${\theta}_{{d}} = {\theta}_{600} - {\theta}_{{w}}$$

6 $${\theta}_{{a}} = {\theta}_{{f }} \, -{ \theta}_{{w}}$$

where *θ*_*r*_ is the soil readily available water content (cm^3^ cm^−3^), *θ*_*f*_ (cm^3^ cm^−3^) is the field capacity, *θ*_600_ (cm^3^ cm^−3^) is the soil volumetric water content at 600 kPa, *θ*_*d*_ (cm^3^ cm^−3^) is the soil delayed available water content, *θ*_*w*_ (cm^3^ cm^−3^) is the wilting point, and *θ*_*a*_ (cm^3^ cm^−3^) is the soil available water content^22^.

The pore size range of soil capillary porosity was 0.03–0.1 mm and that of soil available water porosity was 0.002–0.06 mm^19^). The ranges of water suction of soil capillary porosity and soil available water porosity were 3–10 kPa and 5–150 kPa, respectively^22^. The soil volumetric water content at 3, 5, 10, and 150 kPa was calculated using formula (2). Soil capillary porosity was the soil volumetric water content at 3 kPa minus the soil volumetric water content at 10 kPa, multiplied by 100%. Soil available water porosity was the soil volumetric water content at 5 kPa minus the soil volumetric water content at 150 kPa, multiplied by 100%^22^.

### Statistical analysis

Analysis of variance (ANOVA) was performed using SAS 6.2 for Windows. The significance of treatment effects in each year was determined using the F-test. Multiple comparisons of means were performed using Duncan’s multiple range test^23^ at *P* ≤ 0.05. IBM SPSS statistics 27 and R language were used for principal component analysis (PCA) analysis.

### Plant materials statement

The experiment complied with relevant institutional, national, and international guidelines and legislation.

## Results

### Soil water retention curve

**Figure 1:**
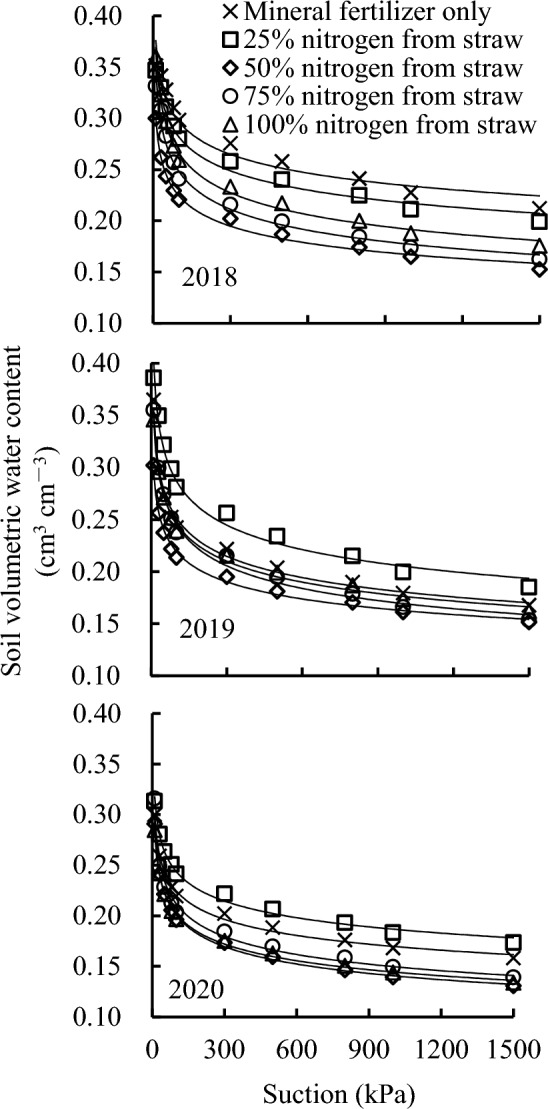
Soil water retention curve as a function of the different replacement treatments in 2018–2020.

The Gardner model^20^ was used to fit the measured data of the soil water retention curve. The points were the measured values, whereas the lines were the fitted values in Fig. 1. The soil water content of each treatment showed a rapid decreasing trend when the water suction was lower than 100 kPa but a slowly decreasing trend when the water suction was greater than 100 kPa.

The fitting coefficient R^2^ of the soil water retention curve of each treatment was above 0.950 (Table 1). The fitting effect was good. Parameter A determined the height of the curve and the level of the water-holding capacity. The larger the value of A, the stronger the water-holding capacity^24^. S_100_ had the highest value of parameter A, followed by S_75_, whereas parameter A with S_25_ and S_50_ was lower than that with CK in 2018. S_25_ had the highest value of parameter A, followed by S_75_, whereas parameter A with S_50_ and S_100_ was lower than that with CK in 2019. S_75_ had the highest value of parameter A, followed by S_25_, S_50_, S_100_, and CK in 2020. Thus, replacement treatments could increase soil water-holding capacity over time.

**Table 1:** Parameters in the modeling of the soil water retention curve as a function of the different replacement treatments in 2018–2020.

Years	Treatments	*A*	*R*^2^
2018	Mineral fertilizer only	0.4765	0.950
	25% nitrogen from straw	0.4742	0.972
	50% nitrogen from straw	0.4047	0.993
	75% nitrogen from straw	0.4872	0.981
	100% nitrogen from straw	0.5129	0.991
2019	Mineral fertilizer only	0.4899	0.987
	25% nitrogen from straw	0.5616	0.985
	50% nitrogen from straw	0.3996	0.993
	75% nitrogen from straw	0.5153	0.993
	100% nitrogen from straw	0.4813	0.995
2020	Mineral fertilizer only	0.3925	0.994
	25% nitrogen from straw	0.4143	0.993
	50% nitrogen from straw	0.4088	0.997
	75% nitrogen from straw	0.4227	0.982
	100% nitrogen from straw	0.3936	0.996

*A* and *B* are dimensionless parameters related to the curve shape.

### Soil specific water capacity

**Figure 2:**
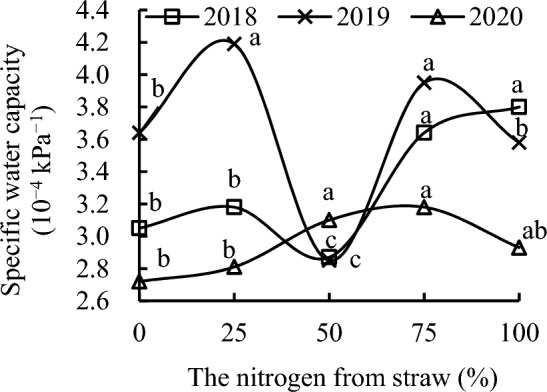
Specific water capacity of soil at 100 kPa as a function of the different replacement treatments in 2018–2020.

The soil specific water capacity at 100 kPa soil water suction reflected soil water supply capacity well^25^. S_50_ decreased soil specific water capacity by 5.93% compared with CK, whereas S_75_ and S_100_ increased it by 19.16% and 24.30%; by 14.28% and 19.21%; and by 26.67% and 32.14% compared with CK, S_25_, and S_50_, respectively, in 2018 (Fig. 2). Soil specific water capacity with S_25_ was 10.84% higher than that with S_50_ in 2018.

S_50_ decreased soil specific water capacity by 21.64% compared with CK. However, S_25_ and S_75_ increased soil specific water capacity by 14.90% and 8.50%, respectively, compared with CK; and by 46.63% and 38.46%, respectively, compared with S_50_; and by 16.95% and 10.43%, respectively, compared with S_100_ in 2019. Soil specific water capacity with S_100_ was 25.38% higher than that with S_50_ in 2019.

Replacement treatments increased soil specific water capacity in 2020. S_50_, S_75_, and S_100_ increased soil specific water capacity by 13.87%, 16.81%, and 7.60%, compared with CK in 2020. Soil specific water capacity with S_50_ and S_75_ was 10.41% and 13.27% higher than that with S_25_, and 5.83% and 8.57% higher than that with S_100_, in 2020.

Thus, the replacement of nitrogen from mineral fertilizer with equivalent nitrogen from maize straw increased soil water supply capacity over time.

### Soil water constant

**Figure 3:**
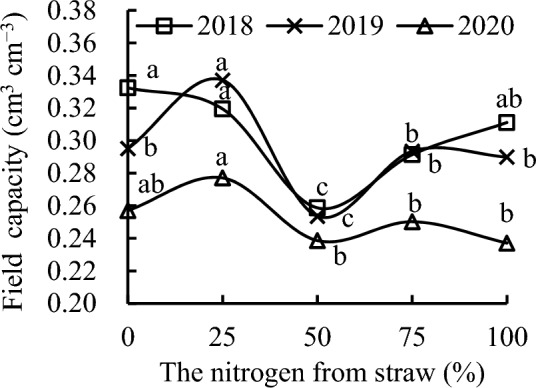
Field capacity as a function of the different replacement treatments in 2018–2020.

Compared with CK, S_25_ slightly decreased field capacity, whereas S_50_, S_75_, and S_100_ decreased it by 22.17%, 12.33%, and 6.41%, respectively, in 2018 (Fig. 3). S_25_ increased field capacity by 14.23% and 7.78%, whereas S_75_ slightly decreased it, and S_50_ decreased it by 14.06% and 7.20% in 2019 and 2020, respectively. S_100_ slightly decreased field capacity in 2019 and decreased it by 7.78% in 2020.

**Figure 4:**
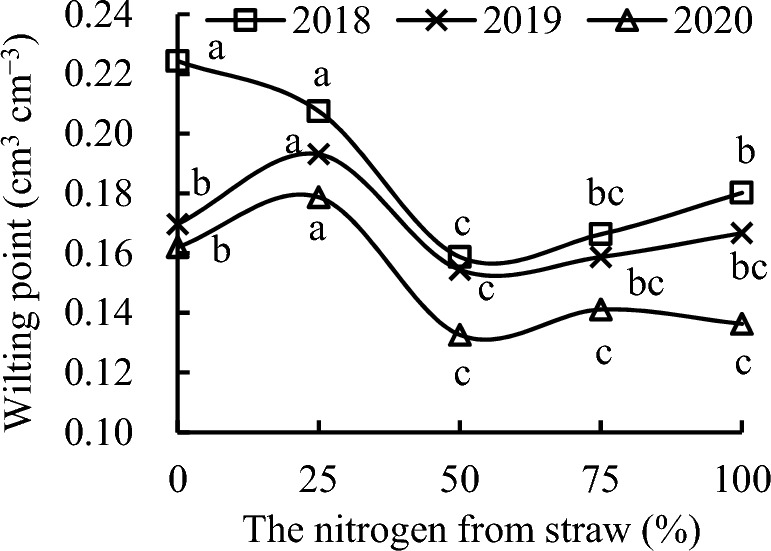
Wilting point as a function of the different replacement treatments in 2018–2020.

S_25_, S_50_, S_75_, and S_100_ decreased the wilting point by 7.49%, 29.25%, 25.86%, and 19.66%, respectively, compared with CK in 2018 (Fig. 4). The wilting point with S_25_ was 13.79% and 10.31% higher than that with CK in 2019 and 2020, respectively. Relative to CK, S_50_ and S_75_ decreased the wilting point by 9.02% and 6.48%, respectively, in 2019, and by 18.15% and 12.90%, respectively, in 2020. S_100_ slightly decreased the wilting point in 2019, and decreased it by 15.86% in 2020 compared with CK.

**Figure 5:**
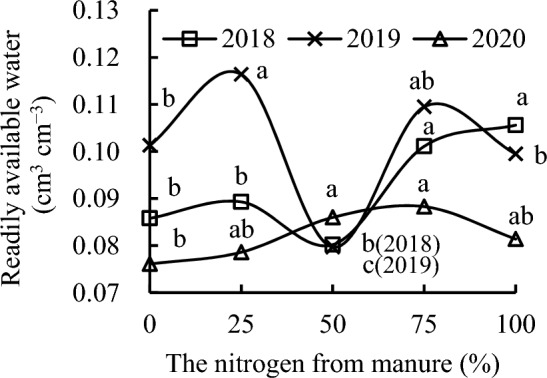
Readily available water content as a function of the different replacement treatments in 2018–2020.

**Figure 6:**
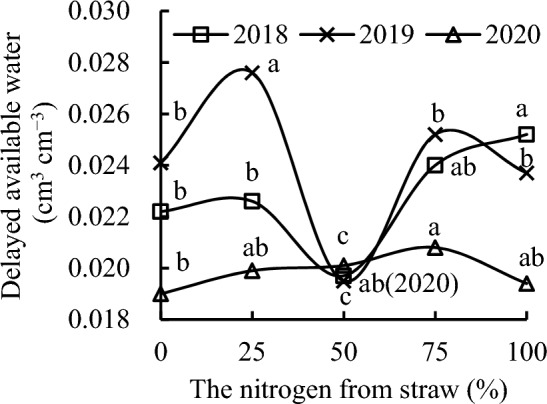
Delayed available water content as a function of the different replacement treatments in 2018–2020.

**Figure 7:**
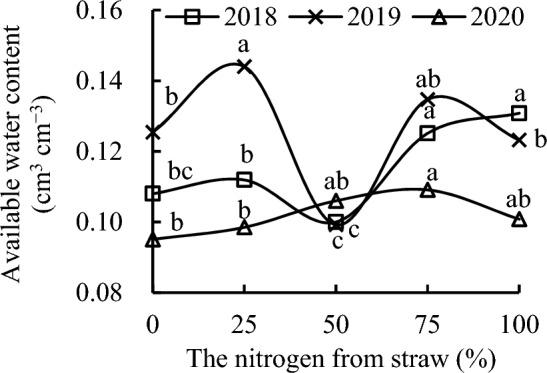
Available water content as a function of the different replacement treatments in 2018–2020.

S_100_ had the highest soil readily available water, soil delayed available water, and soil available water, followed by S_75_ in 2018 (Figs. 5, 6, 7). S_100_ increased soil readily available water, soil delayed available water, and soil available water by 23.08%, 13.51%, and 21.11%, respectively, compared with CK; by 31.67%, 27.92%, and 30.80%, respectively, compared with S_50_; and by 18.25%, 11.50%, and 16.89%, respectively, compared with S_25_ in 2018. S_75_ increased soil readily available water, soil delayed available water, and soil available water by 17.83%, 8.11%, and 15.83%, respectively, compared with CK; by 26.06%, 21.83%, and 25.10%, respectively, compared with S_50_; and by 13.21%, 6.20%, and 11.80%, respectively, compared with S_25_ in 2018. S_25_ increased soil readily available water, soil delayed available water, and soil available water by 11.35%, 14.72%, and 11.90%, respectively, compared with S_50_, and slightly increased these compared with CK, whereas S_50_ decreased these by 6.53%, 11.26%, and 7.41%, respectively, compared with CK in 2018.

S_25_ had the highest soil readily available water, soil delayed available water, and soil available water, followed by S_75_ in 2019. S_25_ increased soil readily available water, soil delayed available water, and soil available water by 14.91%, 14.52%, and 14.83%; by 46.05%, 41.54%, and 45.16%; by 6.30%, 9.52%, and 6.90%; and by 16.98%, 16.46%, and 16.88% compared with CK, S_50_, S_75_, and S_100_, respectively, in 2019. S_75_ increased soil readily available water, soil delayed available water, and soil available water by 8.09%, 4.56%, and 7.42%, respectively, compared with CK; by 37.39%, 29.23%, and 35.79%, respectively, compared with S_50_; and by 10.05%, 6.33%, 9.33%, respectively, compared with S_100_ in 2019. S_50_ decreased soil readily available water, soil delayed available water, and soil available water by 21.32%, 19.09%, and 20.89%, respectively, whereas S_100_ slightly decreased these compared with CK in 2019.

Replacement treatments could increase soil readily available water, soil delayed available water, and soil available water in 2020. S_75_ had the highest soil readily available water, soil delayed available water, and soil available water, followed by S_50_ in 2020. S_75_ increased soil readily available water, soil delayed available water, and soil available water by 16.03%, 9.47%, and 14.72%, respectively, compared with CK; by 12.34%, 4.52%, and 10.76%, respectively, compared with S_25_; and by 8.48%, 7.22%, and 8.23%, respectively, compared with S_100_ in 2020. S_50_ increased soil readily available water, soil delayed available water, and soil available water by 13.01%, 5.79%, and 11.46%, respectively, compared with CK. The soil readily available water, soil delayed available water, and soil available water first increased and then decreased with the increase in nitrogen from straw instead of nitrogen from chemical fertilizer.

### Soil equivalent pore

**Figure 8:**
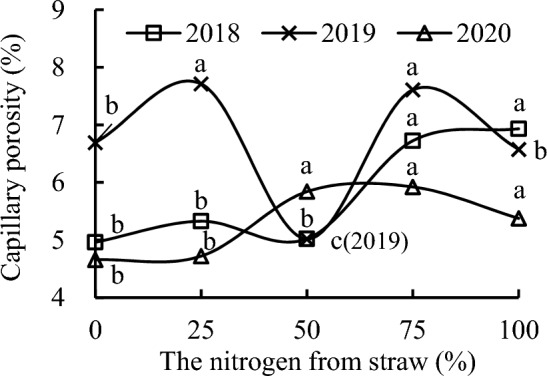
Capillary porosity of soil as a function of the different replacement treatments in 2018–2020.

Compared with CK, S_50_ slightly increased soil capillary porosity, whereas S_25_, S_75_, and S_100_ increased it by 7.35%, 35.49%, and 39.70%, respectively, in 2018 (Fig. 8). S_100_ increased soil capillary porosity by 30.13% and 38.06%, whereas S_75_ increased it by 26.21% and 33.9% compared with S_25_ and S_50_, respectively, in 2018. Soil capillary porosity with S_25_ was 6.09% higher than that with S_50_ in 2018.

S_100_ slightly decreased soil capillary porosity, whereas S_50_ decreased it by 25.00% relative to CK in 2019. S_25_ and S_75_ increased soil capillary porosity by 15.23% and 13.69%; by 53.64% and 51.59%; and by 17.29% and 15.72% compared with CK, S_50_, and S_100_, respectively, in 2019. Soil capillary porosity with S_100_ was 31.00% higher than that with S_50_ in 2019.

S_25_ slightly increased soil capillary porosity compared with CK, whereas S_50_ and S_75_ increased it by 25.39% and 27.14%; by 23.73% and 25.46%; and by 8.65% and 10.17% relative to CK, S_25_, and S_100_, respectively, in 2020. Soil capillary porosity with S_100_ was 15.41% and 13.88% higher than that with CK and S_50_, respectively, in 2020.

**Figure 9:**
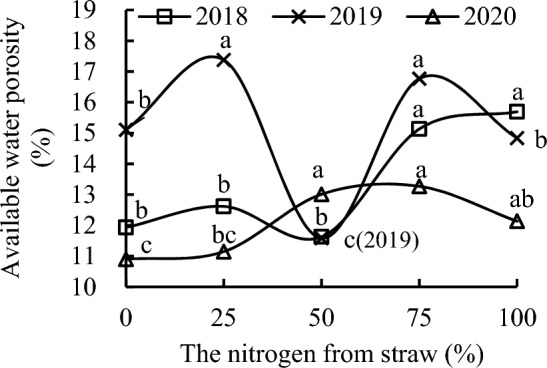
Available water porosity of soil as a function of the different replacement treatments in 2018–2020.

S_50_ slightly decreased soil available water porosity compared with CK, whereas S_25_, S_75_, and S_100_ increased it by 5.73%, 26.81%, and 31.53%, and by 8.52%, 30.15%, and 34.99% compared with CK and S_50_, respectively, in 2018. S_75_ and S_100_ increased soil available water porosity by 19.94% and 24.40%, respectively, relative to S_25_ in 2018 (Fig. 9). Soil available water porosity with S_25_ was 8.52% higher than that with S_50_ in 2018.

S_100_ slightly decreased soil available water porosity, whereas S_50_ decreased it by 23.30% relative to CK in 2019. S_25_ and S_75_ increased soil available water porosity by 15.06% and 11.03%; by 50.01% and 44.76%; and by 17.12% and 13.01% compared with CK, S_50_, and S_100_, respectively, in 2019. Soil available water porosity with S_100_ was 28.09% higher than that with S_50_ in 2019.

S_25_ slightly increased soil available water porosity compared with CK, whereas S_50_ and S_75_ increased it by 19.36% and 21.74%; by 16.72% and 19.05%; and by 7.21% and 9.35% compared with CK, S_25_, and S_100_, respectively, in 2020. Soil available water porosity with S_100_ was 11.33% and 8.87% higher than that with CK and S_25_, respectively, in 2020.

### Principal component analysis

The values of soil water constant, soil available water content, and soil equivalent pore size reflect soil water-holding capacity and soil water supply capacity. To comprehensively measure the indices of soil water constant, soil available water content, and soil equivalent pore size, principal component analysis (PCA) was performed with the evaluation variables of soil specific water capacity (X_1_), field capacity (X_2_), wilting point (X_3_), soil readily available water content (X_4_), soil delayed available water content (X_5_), soil available water content (X_6_), soil capillary porosity (X_7_), and soil available water porosity (X_8_). Principal components (PCs) were extracted according to the criteria of characteristic values greater than 1 (Table 2). The first two PCs had a cumulative contribution rate of 99.97%. Thus, the original eight indices could be replaced by the two PCs for comprehensive evaluation. The PC1 contribution rate reached 80.08%, which mainly reflected the influence of soil specific water capacity (X_1_), soil readily available water content (X_4_), soil delayed available water content (X_5_), soil available water content (X_6_), soil capillary porosity (X_7_), and soil available water porosity (X_8_). The PC2 contribution rate reached 19.88%, which mainly reflected the influence of field capacity (X_2_) and wilting point (X_3_) (Table 3). Thus, soil specific water capacity, soil readily available water, soil delayed available water, soil available water, soil capillary porosity, and soil available water porosity could better reflect soil water-holding capacity and soil water supply capacity compared with field capacity and wilting point.

**Table 2:** Explanation of total variance across principal component analysis.

Principal component	Eigenvalue	Variance contribution rate (%)	Cumulative variance contribution rate (%)
1	6.4066565	80.08	80.08
2	1.5906670	19.88	99.97
3	0.0026057	0.03	100.00
4	0.0000449	0	100.00
5	0.0000194	0	100.00
6	0.0000036	0	100.00
7	0.0000023	0	100.00
8	0.0000005	0	100.00

**Table 3:** Component matrix of principal component.

Indicator variable	Principal component	
	1	2
*X*_1_	0.391	− 0.116
*X*_2_	0.288	0.543
*X*_3_	0.136	0.744
*X*_4_	0.392	− 0.096
*X*_5_	0.393	0.079
*X*_6_	0.394	− 0.065
*X*_7_	0.370	− 0.277
*X*_8_	0.382	− 0.204

**Figure 10:**
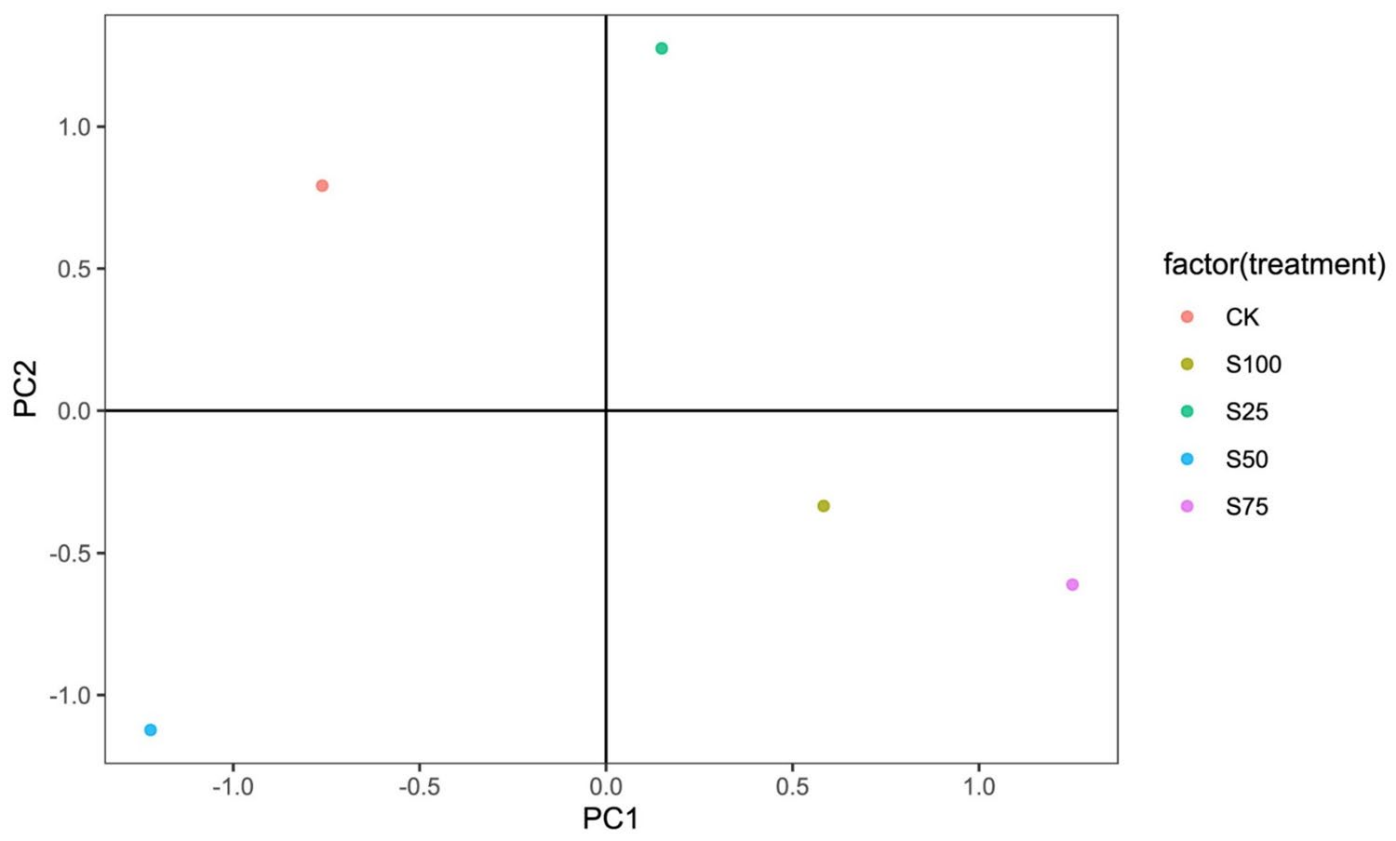
Principal component analysis based on three-year average data.

According to principal component analysis based on three-year average data, S_25_, S_75_ and S_100_ positively correlated with the first principal component (Fig. 10). CK and S_50_ negatively correlated with the first principal component. S_75_ and S_100_ had a close distance, both located in quadrant IV, belonging to the same category. S_25_, CK and S_50_ were far apart, located in quadrants I, II, and III respectively, belonging to different categories.

## Discussion

The soil water characteristic curve can reflect the water holding and releasing characteristics of different soils, and can also be used to understand some soil water constants and characteristic indexes. Therefore, it is an important tool for studying soil water movement, regulating and utilizing soil water, and soil improvement. Fan et al. (2020) Fan^26^ reported that incorporating straw of rape, maize, potato, oats, and buckwheat increased field capacity compared with non-cultivated and without fertilization in the 0–60-cm soil layer. Ren^27^ demonstrated that different straw returning depths enhanced field capacity compared with no fertilization. Du^28^ showed that the wilting coefficient of finely cut straw was higher than long cut straw. In this study, S_25_ increased field capacity and wilting point from the fourth fertilization year, whereas the rest of the replacement treatments had lower field capacity and wilting point compared with CK. This might be because S_25_ had lower straw incorporation rate, which resulted in complete straw decomposition, whereas the rest of the replacement treatments had a higher straw incorporation rate, which resulted in incomplete straw decomposition. In addition, the replacement of mineral fertilization with straw might have immobilized soil inorganic N in the initial stages of maize. Meanwhile, because the decomposition degree of straw was affected by soil moisture, the nitrogen, phosphorus, potassium and other trace elements released during the decomposition of straw would affect the soil moisture and nutrient status and improve the water retention performance of the soil.

Yang^29^ found that the returning of straw of rice, maize, and wheat significantly increased soil water retention capacity at the matric potential of − 0.033 and − 1.5 MPa, and consequently, enhanced soil available water content. Fan^30^ reported that straw returning improved the effects of potassium fertilizer on soil porosity. This study showed that S_50_ decreased soil specific water capacity, soil readily available water, soil delayed available water, soil available water, and soil available water porosity in 2018 and 2019, whereas S_50_ increased these in 2020 compared with single application of mineral fertilizer. These indicated that S_50_ could increase soil water-holding capacity and soil water supply capacity over time. Because the crop root system is the absorption organ of soil moisture and nutrients, it can respond to soil moisture. It was speculated that the reason was that the effective nitrogen content in the soil was sufficient when the content of straw nitrogen and inorganic nitrogen was 50% respectively, which might increase the root density by stimulating the growth of maize roots near the nitrogen-rich area, thereby increase the hydraulic conductivity of maize and improve the absorption of soil moisture by maize. This study also presented that replacement treatments increased soil readily available water, soil delayed available water, soil available water, soil capillary porosity, and soil available water porosity relative to single application of mineral fertilizer in the fifth fertilization year. This might be because continuous straw incorporation promoted the formation of larger soil macroaggregates^31^, which resulted in the improvement of soil structure.

Soil organic carbon is an important chemical component in soil, which can characterize the change in soil quality. Studies had shown that different proportions of straw returning had different effects on the change of soil organic carbon content, but less or too much straw returning would hinder the decomposition of straw and slow down the increased rate of soil organic carbon^32^. At the same time, the application of nitrogen fertilizer could change the number of microbial populations in the soil, promote their activity, build a healthy soil micro-ecological environment, further promote the growth of crop roots, and strengthen the efficient use of water in the soil by crops. Straw returning would also increase the content of soil organic carbon^33^. Soil organic carbon is also the main precursor of soil humus and aggregates, which is of great significance in improving soil fertility. This shows that the application of appropriate straw nitrogen substitution for inorganic fertilizer may be to maintain soil moisture and nutrient characteristics by increasing the content of organic carbon in the soil. Besides, the results showed that soil specific water capacity, soil readily available water, soil delayed available water, soil available water, soil capillary porosity, and soil available water porosity could better reflect soil water-holding capacity and soil water supply capacity compared with field capacity and wilting point. It was indicated that in this experimental area when the straw was used to replace part of chemical fertilizer under the condition of equal nitrogen amount, more attention should be paid to the changes of soil specific water capacity, soil readily available water, soil delayed available water, soil available water, soil capillary porosity and soil available water porosity. Fertilization measures to increase soil specific water capacity, soil readily available water, soil delayed available water, soil available water, soil capillary porosity and soil available water porosity had a beneficial effect on improving soil water status and promoting soil health, thereby promoting crop growth. It could also be speculated that the appropriate adjustment of the proportion of straw nitrogen instead of chemical fertilizer nitrogen according to rainfall conditions would help to better play the benefits of straw returning to the field, promote the water and nitrogen cycle of agricultural ecosystems, and maintain ecological balance.

The significance of replacing part of mineral nitrogen with organic nitrogen in straw is also to reduce the application of mineral nitrogen without damaging the nutritional status of the plant. In this experiment, the effects of straw organic nitrogen instead of mineral nitrogen on soil moisture characteristics in different experimental years were mainly explored. Efficient use of water is one of the important conditions to ensure the normal growth and development of crops. Under the condition of equal nitrogen amount of straw instead of chemical fertilizer application, it can not only ensure the normal metabolic activity in the process of crop vegetative growth and reproductive growth, but also promote the improvement of soil physical and chemical properties, and finally achieve the effect of saving chemical fertilizer and reducing environmental pollution, which is the focus of this experimental study in the future.

## Conclusions

Replacing nitrogen from mineral fertilizer with nitrogen from maize straw gradually increased soil water-holding capacity and soil water supply capacity relative to applying mineral fertilizer only over time. The 25% nitrogen provided by maize straw combined with the 75% nitrogen provided by mineral fertilizer increased field capacity and wilting point compared with applying mineral fertilizer only at the same nitrogen content. Applying equal proportions of nitrogen from maize straw and mineral fertilizer increased soil water availability and soil available water porosity relative to applying mineral fertilizer only from the fifth fertilization year.

## Data Availability

The data that support the findings of this study are available from the corresponding author, upon reasonable request.
